# Bisperoxovanadium promotes motor neuron survival and neuromuscular innervation in amyotrophic lateral sclerosis

**DOI:** 10.1186/s13041-021-00867-7

**Published:** 2021-10-11

**Authors:** Junmei Wang, Lydia Tierney, Ranjeet Mann, Thomas Lonsway, Chandler L. Walker

**Affiliations:** 1 Department of Biomedical Sciences and Comprehensive Care, Indiana University School of Dentistry, Indianapolis, IN 46202 USA; 2 Neuromuscular Research Group, Richard L. Roudebush Veterans Affairs Medical Center, Indianapolis, IN 46202 USA

**Keywords:** Amyotrophic lateral sclerosis, ALS, PTEN, bpV, Akt, Motor neuron

## Abstract

Amyotrophic lateral sclerosis (ALS) is the most common motor neuron (MN) disease, with no present cure. The progressive loss of MNs is the hallmark of ALS. We have previously shown the therapeutic effects of the phosphatase and tensin homolog (PTEN) inhibitor, potassium bisperoxo (picolinato) vanadium (bpV[pic]), in models of neurological injury and demonstrated significant neuroprotective effects on MN survival. However, accumulating evidence suggests PTEN is detrimental for MN survival in ALS. Therefore, we hypothesized that treating the mutant superoxide dismutase 1 G93A (mSOD1^G93A^) mouse model of ALS during motor neuron degeneration and an in vitro model of mSOD1^G93A^ motor neuron injury with bpV(pic) would prevent motor neuron loss. To test our hypothesis, we treated mSOD1^G93A^ mice intraperitoneally daily with 400 μg/kg bpV(pic) from 70 to 90 days of age. Immunolabeled MNs and microglial reactivity were analyzed in lumbar spinal cord tissue, and bpV(pic) treatment significantly ameliorated ventral horn motor neuron loss in mSOD1^G93A^ mice (*p* = 0.003) while not significantly altering microglial reactivity (*p* = 0.701). Treatment with bpV(pic) also significantly increased neuromuscular innervation (*p* = 0.018) but did not affect muscle atrophy. We also cultured motor neuron-like NSC-34 cells transfected with a plasmid to overexpress mutant SOD1^G93A^ and starved them in serum-free medium for 24 h with and without bpV(pic) and downstream inhibitor of Akt signaling, LY294002. In vitro, bpV(pic) improved neuronal viability, and Akt inhibition reversed this protective effect (*p* < 0.05). In conclusion, our study indicates systemic bpV(pic) treatment could be a valuable neuroprotective therapy for ALS.

Amyotrophic lateral sclerosis (ALS) is a devastating fatal neurological disease without a cure. Progressive upper and lower motor neuron (MN) death in the brain and spinal cord are hallmarks of the disease, leading to muscle paralysis and, eventually, respiratory failure. The average prognosis for survival is 3–5 years following diagnosis [1]. Research into the mechanisms of MN loss and methods to promote their survival have been key areas of study for many years, though no truly effective treatments have been discovered. Although ALS is a non-cell-autonomous disease with many dynamic factors influencing MN degeneration, intrinsic mechanisms within the MN are ultimately essential in the process. Evidence over the last several years has highlighted the negative influence of the phosphatase and tensin homolog (PTEN), an antagonist of phosphatidylinositol-3-kinase (PI3K)/Akt signaling on MN vitality in ALS and other MN diseases [2–4]. This is in line with evidence in other neurological diseases and disorders, making PTEN a potentially prime target for therapeutic intervention.

We have previously demonstrated potent neuroprotective effects of PTEN inhibition on MN survival in central nervous system injury models. Systemic treatment with the PTEN antagonist small molecule, potassium bisperoxo (picolinato) vanadium (bpV[pic]) during neurodegenerative phases post-injury reduced the extent of neurological damage, enhanced ventral horn MN survival, and improved vascularization around the site of injury [5]. In addition, our findings demonstrated that bpV(pic) reduced acute neuronal atrophy and upregulated neuronal Akt signaling as a mechanism of its neuroprotective effects [6, 7]. With growing data in support of bpV(pic) as a neuroprotective therapy and accumulating evidence that PTEN may be contributing to MN degeneration in ALS, we proposed to test this therapy in animal and in vitro models of ALS and determine whether similar protective effects are mediated.

Motor neuron degeneration is evident pre-symptomatically between 60 and 90 days of age in the gold standard mutant superoxide dismutase 1 G93A (mSOD1^G93A^) mouse model of ALS (B6.Cg-Tg(SOD1*G93A)1Gur/J #004435; Jackson Laboratory) [8]. In the present study, we administered 400 μg/kg bpV(pic) (Cat. No. SML0885, Sigma-Aldrich) (n = 7) or saline (vehicle) daily (intraperitoneal, i.p.) (n = 7) in mSOD1^G93A^ mice from 70 to 90 days of age followed by euthanasia via i.p. injection of 2,2,2-tribromoethanol (Avertin) (Cat. No. T48402, Millipore Sigma) and transcardial perfusion with 0.1 M phosphate-buffered saline (PBS) and 4% paraformaldehyde (PFA) in PBS. Wild-type littermates were also euthanized for control tissue isolation and preparation (n = 6). The lumbar spinal cord was harvested, cryosectioned cross-sectionally at 20 μm, and serially mounted on microscope slides (Superfrost Plus, Fisher Scientific). Selected lumbar spinal cord sections were incubated with primary rabbit monoclonal antibody against the neuronal nuclear marker, NeuN (1:500, Cat. No. 24307, Cell Signaling, Inc.) and others with rabbit polyclonal Iba-1 antibody (1:1000, Cat. No. 019-19741, Wako Puro Chemical Corp.) overnight for microglial labeling. Fluorophore-conjugated secondary antibody (Jackson Immunoresearch) was applied the following day, and images of the ventral horn were taken of the labeled sections using a Nikon Eclipse Ti epifluorescent microscope and Nikon NIS-Elements software. NeuN-positive neurons in the ventral horn were counted, and the intensity of Iba-1 immunofluorescence was measured in Image J software (NIH).

As neuromuscular junction denervation and atrophy begin prior to symptom onset, the gastrocnemius muscle from mice in each group were harvested and weighed. The muscle was then post-fixed in 4% PFA in PBS for 30 min and then prepared for cryosectioning as described for the spinal cord. The muscle tissue was cryosectioned and incubated with chicken polyclonal antibody against Neurofilament-H (1:500, Cat. No. AB5539, EMD Millipore) overnight at 4 °C in PBST + 10% goat serum. The tissues were then washed and incubated in appropriate secondary antibody and AlexaFluor 594-conjugated α-bungarotoxin (α-BT) for 1 h at room temperature, washed again and coverslip mounted. Five images along the motor end plates in four muscle sections per mouse were collected and total and NF-H/α-BT co-labeled NMJs were quantified in Image J.

We plated NSC-34 cells transfected with human mSOD1^G93A^ plasmid in 96-well plates for imaging of morphology and WST-1 viability (Roche) or lactate dehydrogenase (LDH) release assays. Six-well culture plates were seeded and prepared for cell lysate collection and Western blot analysis. The cells were cultured in growth medium (Dulbecco’s Modified Eagle Medium [DMEM] + 10% fetal bovine serum (FBS, Hyclone) + 1% penicillin/streptomycin (Life Technologies) to approximately 80% confluence. Next, cells plated in both dish types were induced to express mutant SOD1^G93A^, and stress was applied through 24-h serum starvation (DMEM only). During starvation, cells were incubated with either saline (Control), 100 ng/mL bpV(pic) (in both 96- and 6-well plates) or bpV(pic) plus 20 μg/mL PI3K inhibitor, LY294002, in DMEM to determine whether bpV(pic) acted through Akt signaling. The cell lysate was prepared in Laemmli sample buffer with 2-mercaptoethanol, loaded into 4–20% sodium dodecyl sulfate (SDS) polyacrylamide TGX gels (Bio-Rad) for electrophoresis and Western blot analysis. Membranes were incubated overnight with rabbit monoclonal antibody against phospho-Akt (Ser473) (1:1000, Cell Signaling Inc., Cat. No. #4060) and mouse monoclonal antibody against pan-Akt (1:1000, Cell Signaling Inc., Cat. No. 2920).

Cells in 96-well plates were washed in PBS, fixed with 4% PFA, and imaged on an inverted brightfield microscope (Olympus CKX53). Formazan product from the WST-1 assay and colorimetric change from the LDH release assay were analyzed at 420 and 490 nm, respectively, on a Bio-Tek plate reader. Statistical analysis was performed using GraphPad Prism 8.0 software (GraphPad, Inc). The normality of the data was determined using a Shapiro–Wilk test. Data between two groups were analyzed by a two-tailed unpaired Student’s t-test, and data between multiple groups were analyzed by a one-way ANOVA and Newman-Keuls post hoc test. Group differences were considered statistically significant when *p* < 0.05.

Quantification of MNs in the lumbar spinal cord ventral horn sections demonstrates the significant loss of MNs by day 90 with normal disease progression in mSOD1^G93A^ mice (Fig. 1B and D). Furthermore, both visual observation and quantitative analysis highlights the significant reduction in MN loss following 20 days of systemic bpV(pic) treatment, especially in the most ventral region of the gray matter (Rexed Lamina IX). Recent work by Tung et al. [9] showed that dysregulation of miR-17 in the mSOD^G93A^ mouse leads to increased PTEN localization, which was associated with MN death and disease progression while replacing miR-17 prevented these effects. As such, it may be that PTEN phosphatase activity may only be part of PTEN’s negative influence on MN survival, and dual targeting of PTEN expression/localization and phosphatase activity could lead to even better outcomes. Given that the present study only targeted PTEN phosphatase activity, it cannot be ruled out that other functions of PTEN could be contributing to our observed results.

**Fig. 1:**
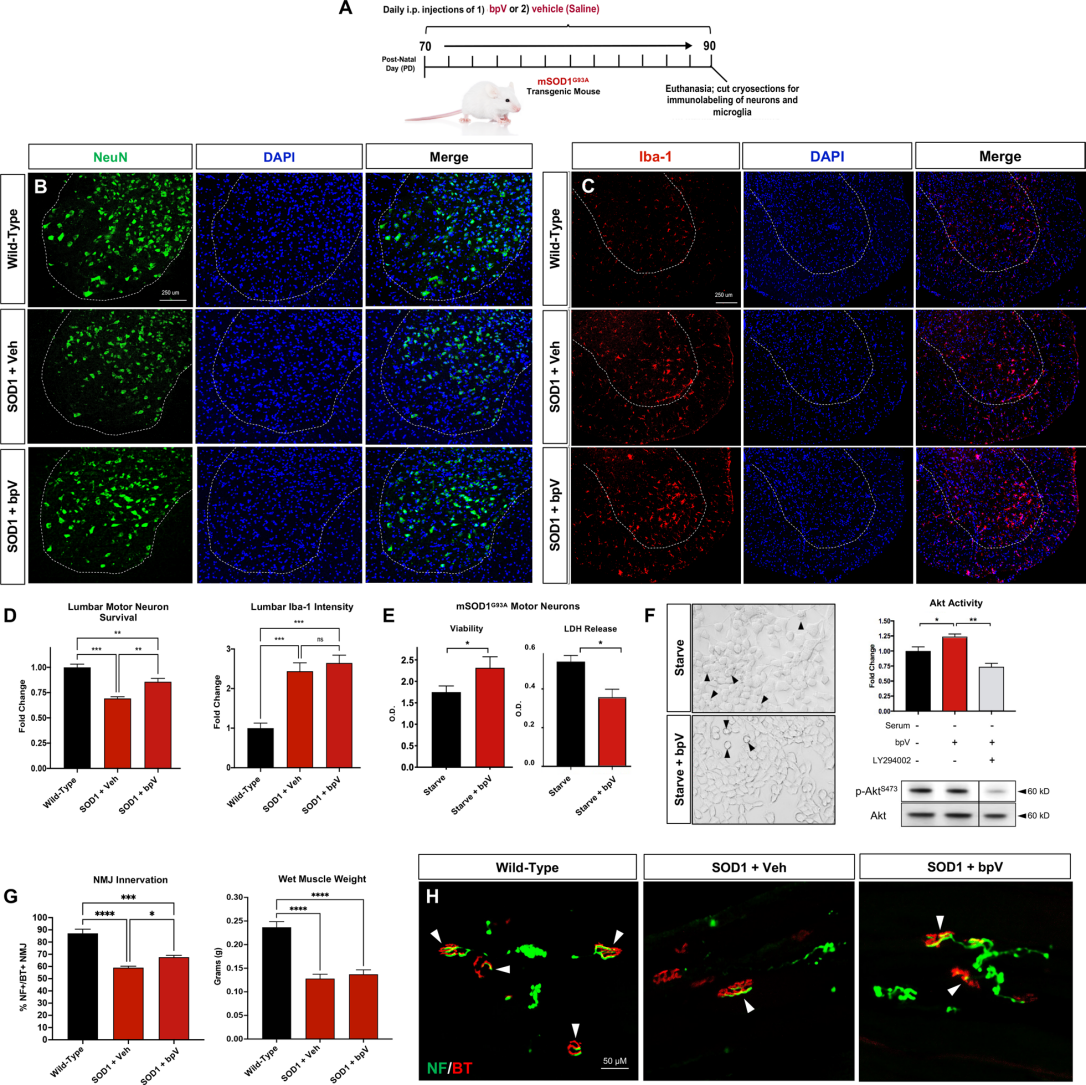
Treatment with bpV(pic) spares MNs in both in vivo and in vitro models of ALS. **A** The treatment schedule and experimental design for in vivo therapy of mSOD1^G93A^ ALS mice. **B** and **D** Ventral horn motor neurons were significantly less than wild-type (WT) mice at 90 days of age (*p* < 0.001). A late pre-symptomatic daily treatment schedule between days 70 and 90 of age enhanced the number of NeuN-positive motor neurons (*p* = 0.003) but did not significantly alter Iba-1-positive microglial reactivity (*p* = 0.701) in the ventral horn of the lumbar spinal cord in mSOD1^G93A^ mice compared to vehicle-treated mice (**C** and **D**). **E** bpV(pic) improved morphology and significantly enhanced viability (*p* = 0.018) and reduced LDH release (*p* = 0.025) in 24-h serum-starved mSOD1^G93A^-expressing motor neuron cells. F) In addition, bpV(pic) significantly increased p-Akt^S473^/Akt ratio following treatment (*p* < 0.05) while co-culture with LY294002 under starvation conditions blocked bpV(pic)’s stimulating effects (*p* < 0.01). G & H) Gastrocnemius muscle assessment showed NMJs were innervated significantly more in bpV(pic)-treated mice than vehicle control mice (*p* = 0.018), but the treatment did not significantly affect muscle size and weight. *ALS* amyotrophic lateral sclerosis (ALS); *NeuN* neuronal nuclear antigen; mSOD1^G93A^ Mutant superoxide dismutase 1 G93A; *LDH* lactate dehydrogenase; *NMJ* neuromuscular junction. WT group, n = 6; vehicle and bpV(pic) groups, n = 7 each). Scale bars = 250 μM (**B** and **C**) and 50 μM (**H**). All cell culture experiment data are from experiments performed in triplicate

The bpV(pic) treatment paradigm did not exacerbate microglial activity in the ventral horn surrounding MNs (Fig. 1C and D), suggesting that either microglial reactivity is not detrimental to MN health at this stage in disease progression or that bpV(pic) may alter reactive microglia toward a more anti-inflammatory (M2) versus pro-inflammatory (M1) phenotype. Data support the latter, as PTEN exhibits a prohibitory effect on M1 to M2 macrophage transition [10]. We, however, did not examine M1 vs. M2 microglial phenotype to confirm whether PTEN inhibition by bpV(pic) modifies microglial phenotype in this way.

Our findings also indicated peripheral therapeutic effects of bpV(pic) therapy in mSOD1^G93A^ mice. Specifically, significantly more neuromuscular junctions (NMJs) were innervated in bpV(pic) treated mice compared to vehicle-treated controls (Fig. 1G and H). It may be that central influence on MN soma as well as peripheral axonal and neuromuscular interaction were all imparted by the treatment. However, with building evidence of an increasingly underappreciated role of skeletal muscle on NMJ reinnervation and repair [11], it is possible the neuronal effects are secondary to target muscle alterations. When we measured wet weight of the gastrocnemius as a measure of treatment effects on muscle atrophy, no significant difference was found compared to vehicle-treated mice (Fig. 1G). This suggests that metabolic changes in muscle may not have been significantly influenced by bpV(pic), but rather a combination of peripheral and central effects contributed to improved neuromuscular innervation. The present study did not investigate longer term effects on survival or function, which would aid in determining whether the increased innervation at 90 days of age leads to a delay of symptom progression and extension of lifespan.

When mSOD1^G93A^-overexpressing NSC-34 cells were stressed with serum starvation for 24 h in the presence of bpV(pic), cell viability was significantly increased while cytotoxicity was significantly downregulated compared to control cells (Fig. 1E and F). As these findings indicate bpV(pic) imparts MN neuroprotection in both in vivo and in vitro mSOD1^G93A^ MNs, we anticipate that our results that PTEN inhibition and Akt activation in the mSOD1^G93A^ motor neuron cell line (Fig. 1F) is a likely mechanism of action for bpV(pic)’s protective effects observed in vivo. However, additional mechanistic investigation in the mSOD1^G93A^ mouse model will be required to test this hypothesis and validate our expected results.

This study is the first to demonstrate the therapeutic effects of bpV compounds through in vivo and in vitro experiments in mSOD1^G93A^ models of ALS. It must be noted that limitations concerning the time frame of investigation of disease status (pre-symptomatically only) and the relatively small sample size of the study impact broader implications of bpV(pic) therapy in altering disease course in later stages of disease. Nevertheless, our findings support prior reports that a primary mechanism of action of bpV(pic) is through inhibition of PTEN phosphatase activity and subsequent upregulation of downstream PI3K/Akt signaling [12, 13]. With evidence mounting for a role of PTEN in MN degeneration in ALS, our evidence of neuroprotective and neuromuscular effects of bpV(pic) on MNs in ALS is promising and provides a new avenue of hope and research into potential effective therapies for preventing MN loss, paralysis, and death in ALS. With increasing support for neuroprotective and regenerative effects of PTEN inhibition, the more confidence that is established in bpV(pic) as a viable and valuable treatment option for ALS.

## Data Availability

Data from the current study are available from the corresponding author on reasonable request.
